# Potential perioperative advantage of colorectal endoscopic submucosal dissection versus laparoscopy-assisted colectomy

**DOI:** 10.1007/s00464-014-3705-5

**Published:** 2014-07-19

**Authors:** Fumihiko Nakamura, Yutaka Saito, Taku Sakamoto, Yosuke Otake, Takeshi Nakajima, Seiichiro Yamamoto, Yoshitaka Murakami, Hideki Ishikawa, Takahisa Matsuda

**Affiliations:** 1 Endoscopy Division, National Cancer Center Hospital, 5-1-1 Tsukiji, Chuo-ku, Tokyo, 104-0045 Japan; 2 Digestive Disease Center, Kohseichuo General Hospital, Tokyo, Japan; 3 Colorectal Surgery Division, National Cancer Center Hospital, Tokyo, Japan; 4 Department of Surgery, Hiratsuka City Hospital, Kanagawa, Japan; 5 Department of Health Science, Shiga University of Medical Science, Shiga, Japan; 6 Department of Molecular-Targeting Cancer Prevention, Kyoto Prefectural University of Medicine, Kyoto, Japan

**Keywords:** Colon, Early colorectal cancer, Endoscopic submucosal dissection, Laparoscopy-assisted colectomy, Quality of life

## Abstract

**Background:**

Endoscopic submucosal dissection (ESD) has recently provided a new treatment strategy for large colorectal neoplasms, as an alternative to laparoscopy-assisted colectomy (LAC). Prospective comparative data on the perioperative course of ESD vis-à-vis LAC are scarce.

**Methods:**

We prospectively evaluated the perioperative course of colorectal ESD in 300 patients. We evaluated *en bloc* and curative resection, procedure duration, postoperative parameters [white blood cell count (WBC), C-reactive protein (CRP), and hemoglobin], pain, recovery duration (time to achieve full mobilization, normal diet, and length of hospitalization), and complications. We also prospectively evaluated 190 patients undergoing LAC as a control group.

**Results:**

The median size of the lesions was 30 mm for ESDs (LACs: 20 mm). The median procedure time was 90 min for ESDs (LACs: 185 min). Postoperative pyrexia was reported in 4 % of ESDs (LACs: 54 %). Only 4 % of ESDs required analgesia (LACs: 61 %). Between the preoperative period and postoperative day 1, the mean difference in WBC and CRP was +1,300/μl for ESDs (LACs: +3,100/μl), and +0.91 mg/dl for ESDs (LACs: +3.96 mg/dl), respectively. A ≥2 g/dl decrease in hemoglobin was observed in 5 % of ESDs (LACs: 30.0 %). Complications were seen in 7 % of ESDs (LACs: 15 %). The rate of delayed bleeding and perforation was 5 and 1.7 % of ESDs, respectively. Although only one of them required laparotomy for peritonitis caused by delayed perforation, others could be managed endoscopically. Additional LAC was required in 16 ESDs due to redefined risk for lymph node metastases. The median hospital stay was 5 days for ESDs (LACs: 10 days). These were consecutive patients with prospective data collection.

**Conclusions:**

Colorectal ESD is effective, minimally invasive and safe in terms of periperative clinical course. Colorectal ESD provides advantages for treatment of large adenomas and early cancers with no risk of lymph node metastasis.

Conventional endoscopic mucosal resection (EMR) is technically inadequate for *en bloc* resection of early colorectal cancer ≥20 mm. Endoscopic piecemeal mucosal resection is associated with a high incidence of local recurrence or suboptimal estimation of pathological invasion depth [1–3].

Endoscopic submucosal dissection (ESD) was initially developed for early gastric cancer and facilitates the resection of large superficial tumors *en bloc* despite the lesion size [4–8]. The introduction of ESD, consequently, has enabled effective treatment of large colorectal tumors and local recurrent lesion after EMR that would previously have been treated by laparoscopy-assisted colectomy (LAC), not only in Asian countries such as Japan, Korea and China, but also in several western countries [9–16] (Figs. 1, 2, 3, and 4). Colorectal ESD, however, can take longer to perform than EMR, according to the location or size of the lesions. ESD is, in addition, technical difficult and may also carry the risk of perforation or peritonitis due to the thin muscularis layer of the colon [17].

**Fig. 1:**
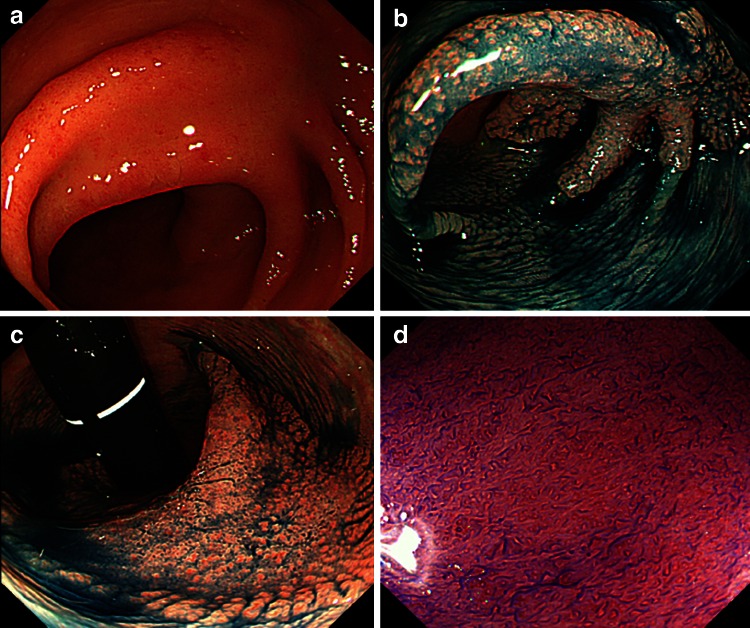
Endoscopic diagnosis before ESD (Case 1). **A** A 0-IIa + IIc non-granular type laterally spreading tumor (LST-NG) 70 mm in size located in the transverse colon. **B**, **C** Lesion margins delineated before ESD using 0.4 % indigo–carmine spray dye. **D** Magnification colonoscopy with crystal violet (0.05 %) staining clearly revealed III_s_ and III_L_ pit patterns in the depressed area, suggesting a non-invasive tumor and indicating a good candidate for endoscopic treatment

**Fig. 2:**
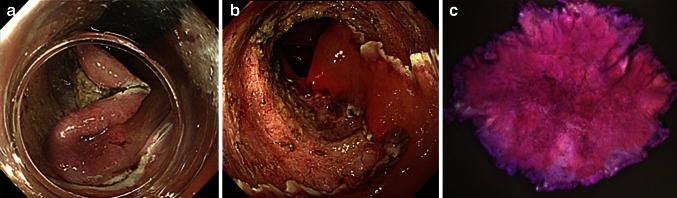
Images of colonic ESD (Case 1). **A** After injection of glycerol (10 % glycerol and 5 % fructose in normal saline solution) and sodium hyaluronate acid solution into SM layer, partial circumferential incision performed by using bipolar needle knife. SM dissection performed by using a bipolar needle knife and insulation-tipped knife. **B** *En bloc* resection was completed. **C** Histology of resected specimen 70 × 55 mm in diameter revealed intramucosal cancer with tumor-free margin

**Fig. 3:**
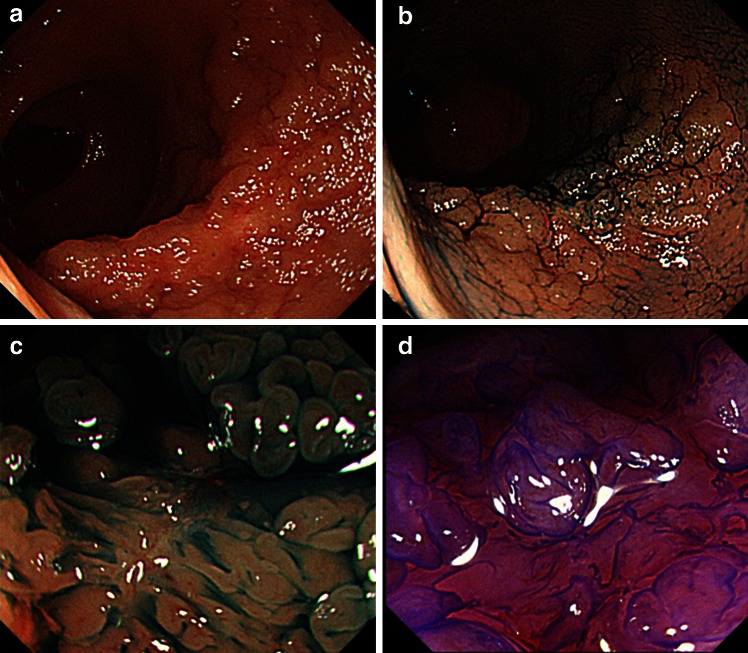
Endoscopic diagnosis before ESD (Case 2). **A** A recurrent tumor was identified at the scar site of a previous endoscopic mucosal resection in the lower rectum. **B** Lesion margins delineated using 0.4 % indigo–carmine spray dye. **C** Magnification colonoscopy with indigo–carmine dye revealed scarring and non-invasive IV pit pattern in this lesion. **D** Crystal violet (0.05 %) staining revealed IV pit pattern suggesting non-invasive tumor and indication of ESD

**Fig. 4:**
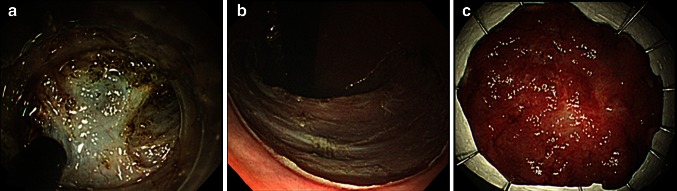
Images of rectal ESD (Case 2). **A** ESD was performed. Marked fibrosis was observed during the procedure. **B** ESD was completed without any complications. **C** Histology of resected specimen (*en bloc* resection) 60 × 40 mm in diameter revealed intramucosal cancer with tumor-free margin

In many cases outside Japan, LAC, which is less invasive than open surgery, is still performed even if the lesion is a good indication for colorectal ESD. Such lesions include adenoma, with mucosal or shallow submucosal (SM) invasion <1,000 µm from the muscularis mucosae (SM-s), with negligible risk of lymph node metastasis. The standard techniques and safety of LAC have been established, and especially in the colon, it is less invasive and does not adversely affect postoperative quality of life (QOL).

Colorectal ESD, in contrast to LAC or open colectomy, allows intraoperative management by utilizing conscious sedation (midazolam or pentazocine hydrochloride), and does not require general anesthesia with intratracheal intubation. We can, therefore, avoid the risk associated with general anesthesia. Patients who undergo colorectal ESD, in addition, can start walking soon after treatment and achieve early recovery of physical ability. These patients can also resume food intake in the early stage because intestinal tract anastomosis is not required for ESD. Another key factor is that anorectal function can be absolutely preserved in rectal ESD.

We have previously reported that rectal ESD significantly decreases the incidence of local recurrence and preserves postoperative QOL, compared with transanal resection [18]. In Korea, a comparison between ESD and transanal endoscopic microsurgery (TEM), the former was less invasive, although there was no significant difference in clinical outcome between the groups [19]. Rectal ESD has recently been introduced in western countries too [20].

In some industrialized institutions or hospitals, ESD is performed for rectal as well as colonic neoplasms and there are many reports of good safety and clinical outcomes for colorectal ESD.Kiriyama and Saito et al. reported good clinical outcomes for colorectal ESD compared with LAC [21]. There are, however, no data to compare the perioperative clinical course after colorectal ESD and LAC, although several prospective studies on colorectal ESD have been reported. It is important to clarify objectively the invasiveness and safety of colorectal ESD as well as its effectiveness.

The primary indication for colorectal ESD used as a local treatment without lymph node dissection is non-invasive lesions diagnosed as adenoma, mucosal, or SM-s colorectal cancer. The absolute indication for LAC with lymph node dissection is T1 colorectal cancer with deep invasion of the submucosa (SM-d). There are different indications and procedures for the two methods and simple comparison is not possible. Considering the present position, in which LAC is still performed instead of colorectal ESD for lesions with less than SM-s invasion in the world, we decided to compare colorectal ESD with LAC for T1 cancer. We prospectively evaluated the perioperative clinical course of colorectal ESD and LAC. Preoperative diagnostic accuracy was also calculated.

## Materials and methods

### Patients

We prospectively enrolled ESD patients diagnosed with adenoma or T1 cancer with less than SM-s invasion at the preoperative conference of the Endoscopy Division, National Cancer Center Hospital (NCCH) in Tokyo, Japan, from January 2009 to March 2012. We also prospectively enrolled a control group at the preoperative joint conference between Endoscopy Division and Colorectal Surgery Division in NCCH. This comprised patients who underwent LAC for SM-d cancer (cT1) diagnosed endoscopically or pathologically after endoscopic treatment. Patients who underwent ESD in our institute have the possibility of requiring additional LAC after histological examination of the excised sample. Since this research is observational study and the process until LAC is completed in such case is a series of clinical course on the basis of colorectal ESD, such cases were decided to be included in the ESD group in this study. All patients gave written informed consent for the endoscopic and surgical resections prior to treatment. This study was performed in accordance with the Helsinki Declaration.

### Exclusion criteria

Exclusion criteria were: (1) non-neoplastic polyps; (2) familial adenomatous polyposis; or (3) submucosal tumor.

### Preoperative diagnosis

Preoperative diagnosis was performed by colonoscopy with magnifying function (CF-HQ290I, CF-H260AZI, PCF-Q240ZI, or PCF-Q260AZI; Olympus Medical Systems Corp., Tokyo, Japan). All lesions were subjected to pit pattern analysis by magnifying chromoendoscopy (MCE) with indigo–carmine dye (0.4 %) and narrow-band imaging (NBI) system. If type V pit pattern was suspected, we also performed estimation by MCE using crystal violet staining (0.05 %). Invasive pattern is used as an index for SM-d cancer [25].

### ESD procedure

ESD was carried out with a PCF-Q240JI, PCF-Q260AZI or GIF-Q260J endoscope (Olympus Medical Systems Corp., Tokyo, Japan) equipped with a short type small caliber-tip transparent hood (Fujifilm Corp., Tokyo, Japan) fitted to the tip of the endoscope to retract the SM layer, thereby facilitating dissection. The procedures were primarily performed using a bipolar needle knife (Xeon Medical Corp., Tokyo, Japan) and insulation-tipped knife (Olympus Medical Systems Corp., Tokyo, Japan). Midazolam (2 mg intravenously) and pentazocine hydrochloride (15 mg intravenously) were administered during all ESD procedures. An additional 2 mg midazolam was given as necessary whenever indicated based on the judgment of the colonoscopist. Bipolar hemostatic forceps (Pentax Corp., Tokyo, Japan) were used for hemostasis of bleeding. CO_2_ insufflation was used instead of air insufflation to reduce patient discomfort. Lesion margins were delineated before ESD using 0.4 % indigo–carmine spray dye. After injection of 10 % glycerol and 5 % fructose in normal saline solution (glycerol; Chugai Pharmaceutical Corp., Tokyo, Japan) and sodium hyaluronate acid (Muco-up; Johnson & Johnson Corp., Tokyo, Japan) into the SM layer, a circumferential incision was made using a bipolar needle knife, and ESD was the performed using one or two ESD knives.

### LAC procedure

One of three expert colorectal surgeons performed LAC according to standard procedures with the patient under general anesthesia. Four trocar incisions were used, which were followed by resection of the diseased colon and rectum, a D2 lymph node dissection, and functional end-to-end anastomosis in the colon or double-stapling anastomosis in the rectum. In some patients with tumors located in the lower rectum, temporary ileostomies were performed as the surgeon judged appropriate.

### Histopathological assessment

The endoscopically resected specimens, after being fixed in formalin, were sectioned serially at 2–3 mm intervals and the surgically resected specimens at 4–5 mm intervals. Histological diagnosis was based on the Japanese Research Society for Cancer of the Colon and Rectum (JSCCR) and the Vienna classification. Invasion depth of early colorectal cancer was subclassified as adenoma, pTis (M), pT1a (SM-s), pT1b (SM-d) or defined as category 5.1 (intramucosal carcinoma) or category 5.2 (SM carcinoma) according to the Vienna classification of gastrointestinal epithelial neoplasia. The depth of SM tumor invasion was measured between lower muscularis mucosae and the tumor invasive top. If the muscularis mucosae were disrupted by tumor invasion, the depth was measured from the surface of the tumor. In the SM carcinoma, the depth of SM invasion which was <1,000 µm was diagnosed as pT1a (SM-s), while one which was more than 1,000 µm was diagnosed as pT1b (SM-d) in accordance with the JSCCR guideline [22].

### Postoperative analysis

We analyzed (1) operation duration, *en bloc* resection and curative resection; (2) postoperative pyrexia requiring analgesic drugs (non-steroidal anti-inflammatory drugs, pentazocine hydrochloride, or fentanyl), early laboratory investigation, hospitalization, early resumption of normal activities such as walking, drinking and eating; and (3) any complications associated with the procedure.

Curative resection was defined as free margins, SM invasion <1,000 µm from the muscularis mucosae without lymphovascular invasion, a poorly differentiated adenocarcinoma component [22]. ESD procedure time was measured from the initial incision to complete removal of the tumor. LAC procedure time was calculated from the start of the operation to its completion. Analgesic use was related to the presence of postoperative pain, and consisted of non-steroidal anti-inflammatory drugs, pentazocine hydrochloride, or fentanyl. We examined the patients on morning and evening ward rounds as well as reference of their charts to register it prospectively. We validated the results of early laboratory investigations. The mean differences in white blood cell count (WBC) and C-reactive protein (CRP) level between the preoperative period and postoperative day (POD) 1 were calculated. Decreases in hemoglobin level of ≥2 g/dl were noted. We assessed patients for resumption of normal activities such as walking, fluid and food intake, and hospital stay, at round visits and from their chart records. We analyzed complications associated with the procedures. In the ESD group, perforation, penetration, peritonitis, and delayed bleeding were assessed prospectively as major complications and anesthesia complications as minor complications. In the LAC group, major complications (anastomotic leakage, peritonitis, and delayed bleeding) and minor complications (ileus, wound infection and dehiscence, and anesthesia complications) were also analyzed prospectively. The rate of diverting stoma due to postoperative adverse event (perforation, anastomotic leakage, or delayed bleeding) was also considered as a major complication in both groups. Total cases of stoma, in addition, were analyzed.

Preoperative endoscopic diagnostic accuracy was also calculated, excluding the cases in which accurate estimation of histopathological invasion depth of resected specimen were difficult. Accordance between preoperative endoscopic estimation and histopathological invasion depth (M-SM-s or SM-d) was defined as proper diagnosis.

### Statistical analysis

Statistical differences were analyzed using the Wilcoxon signed-rank test, Mann–Whitney *U* test, and *χ* ^2^ test. A two-tailed *P* value below 0.05 was considered statistically significant. Statistical analysis was performed using SPSS for Windows, version 10.1 (SPSS, Chicago, IL, USA).

## Results

### Patient characteristics

We excluded three cases of non-neoplastic polyps in the ESDs; three cases of familial adenomatous polyposis in the LACs; one case of submucosal tumor in the ESDs. Moreover, two cases in the ESDs for which postoperative information, including blood analysis data, was lacking were excluded in this study term. This left a final total of 300 ESDs that were included in the study. Among these, 16 required additional LAC based on pathological assessment after ESD: one case with SM-s carcinoma with lymphovascular invasion and 15 with SM-d carcinoma.There were 190 cases enrolled in the LACs.

The median age was 68 years in the ESDs (range 36–98 years) and 65 years (20–86 years) in the LACs. The male to female ratio was 1.1:1 in both groups. The median tumor size was 30 mm (range 8–110 mm) in the ESDs and 20 mm (8–150 mm) in the LACs (Table 1).

**Table 1:** Clinical characteristics of patients

	No. (%) ESDs	No. (%) LACs
Number	300 (ESD + LAC: 16)	190
Age, median (range), y	68 (36–98)	65 (20–86)
Male	157 (52.3)	101 (53.2)
Size, median (range), mm	30 (8–110)	20 (8–150)
Location (rectum/colon)	83/217 (27.7/72.3)	33/160 (17.4/82.6)

In the ESDs, 232 patients (77.3 %) had laterally spreading tumors (LSTs); these comprised 140 granular LSTs (LST-G) (46.7 %) and 92 non-granular LSTs (LST-NG) (30.7 %). In the LACs, there were 106 non-LSTs (55.8 %) and 19 LSTs (11 LST-G and 8 LST-NG) (10.0 %). Sixty-five (34.2 %) of the operations in the LACs were for post-EMR lesions because of non-curative resection. Twenty (6.7 %) of all ESDs were for local recurrence after previous endoscopic resection. Three cases (1.6 %) of local recurrence required LAC. Two of these were diagnosed as SM-d carcinoma by preoperative colonoscopy at NCCH. The other had lymph vessel invasion diagnosed by histological examination of the resected specimen at the referring hospital.

For histopathological diagnosis, 276 lesions (92.3 %) were adenoma or mucosal to SM-s carcinomas, and 21 lesions (7.0 %) were SM-d carcinomas. Although seven of 21 cases of SM-d carcinomas were suspected at preoperative colonoscopy, ESD was carried out diagnostically in accordance with the age, general status, and wish of the patient. In the LACs, 154 (81.0 %) of all cases were SM-d carcinomas and 34 (17.9 %) were mucosal to SM-s carcinomas. In 34 mucosal and SM-s carcinomas further analysis revealed that 13 cases had lymphovascular infiltration or positive vertical margin (pVM) on pathological analysis after EMR in our hospital or the referring hospital. Four cases did not have an indication for ESD because of invasion to the appendix orifice, and two cases underwent LAC at the outset at the patients’ request. Eight patients were selected for LAC because of large protruding lesions (Paris type 0-Is, >40 mm diameter) such as villous tumors, which were likely to be indicated for multiple piecemeal resection or perforation on preoperative endoscopic examination. Only seven cases were mucosal to SM-s carcinomas, although these were suspected of SM-d carcinomas on preoperative colonoscopy. In two (0.67 %) of the ESDs and two (1.1 %) of the LACs, pathological evaluation was difficult even after retrospective reviews (Table 2).

**Table 2:** Tumor features (LSTs versus non-LSTs) and pathological tumor depth

	No. (%) ESDs (*n* = 300)	No. (%) LACs (*n* = 190)
LSTs	232 (77.3)	19 (10.0)
Granular	140	11
Non-granular	92	8
Non-LSTs	68 (22.7)	106 (55.8)
Protruding	21	24
Depressed	27	79
Local recurrence	20	3
Scar from previous EMR	–	65 (34.2)
Pathological tumor depth		
M-SM-s	277 (92.3)	34 (17.9)
SM-d	21 (7.0)	154 (81.0)
Unknown	2 (0.67)	2 (1.1)

*LST* laterally spreading tumor, *EMR* endoscopic submucosal resection, *M* mucosa, *SM*-*s* submucosal invasion <1,000 μm from the muscularis mucosae, *SM*-*d* submucosal invasion 1,000 μm or more from the muscularis mucosae

### Preoperative endoscopic diagnostic accuracy

In two cases in the ESDs, we were unable to obtain accurate pathological information. Sixty-five cases in the LACs (2 of which also had difficult pathological examination) had scarring from previous EMR. All these cases were excluded from the present analysis.

Preoperative endoscopic diagnostic accuracy on the depth of invasion was 95 % (282/298) in the ESDs and 93 % (116/125) in the LACs. Overall diagnostic accuracy was 94 % (398/423). In the ESDs, 11/298 (3.7 %) cases had additional surgery, because of preoperative estimation of shallow invasion depth. In the LACs, 7/125 (5.6 %) cases turned out to be over surgeries, because of preoperative estimation of deep invasion.

### Procedure time and en bloc and curative resection

The median procedure duration in the ESDs was 90 min (range 15–540 min) compared with 185 min (48–499 min) in the LACs (*P* < 0.001). The *en bloc* and curative resection rates in the ESDs were 91.7 % (275/300) and 91.0 % (273/300), respectively (Table 3).

**Table 3:** Clinical outcomes: effectiveness (procedure time, en bloc, and curative resection)

	No. (%) ESDs (*n* = 300)	No. (%) LACs (*n* = 190)	*P* value
Procedure time, median (range), min	90 (15–540)	185 (48–449)	<0.001
En bloc resection	275 (91.7)	–	
Curative resection^a^	273 (91.0)	–	

^a^Curative resection : free margin, submucosal invasion with <1,000 μm from muscularis mucosae without lymphovascular invasion, a poorly differentiated component

### Postoperative pyrexia and early laboratory investigations

The rate of postoperative pyrexia (≥38 °C) was 4.3 % (13/300) in the ESDs, compared with 54.2 % (103/190) in the LACs (*P* < 0.001). The number of patients requiring analgesic drugs because of postoperative, abdominal or wound pain was 13/300 (4.3 %) in the ESDs and 115/190 (60.5 %) in the LACs (*P* < 0.001). In the early laboratory investigations, the mean difference in WBC and CRP between the preoperative stage and POD 1 was +1,300/μl in the ESDs and +3,100/μl in the LACs (*P* < 0.001) and +0.90 mg/dl in the ESDs and +3.96 mg/dl in the LACs (*P* < 0.001), respectively. In the ESDs, only 11/300 (3.6 %) had decrease in hemoglobin level of ≥2 g/dl, compared with 57/190 (30.0 %) in the LACs (*P* < 0.001). No transfusions were required the in ESDs, but they were required in the LACs (5/190; 2.6 %) (*P* = 0.005) (Table 4).

**Table 4:** Clinical outcomes: less invasiveness (pyrexia, requirement for analgesic drugs, early laboratory investigations, hospitalization, and early resumption of normal activities)

	No. (%) ESDs (*n* = 300)	No. (%) LACs (*n* = 190)	*P* value
Postoperative pyrexia (≥38 °C)	13 (4.3)	103 (54.2)	<0.001
Requiring analgesic drugs^a^	13 (4.3)	115 (60.5)	<0.001
Mean variable value of WBC (Pre-op stage/POD1), μl	1,300 (5,900/7,200)	3,100 (5,400/8,500)	<0.001
Mean variable value of CRP (Pre-op stage/POD1), mg/dl	0.90 (0.13/1.04)	3.96 (0.12/4.08)	<0.001
Rate of drop (≥2 mg/dl) in Hb value	15 (5.0)	57 (30.0)	<0.001
Transfusion (RCC-LR)	0 (0)	5 (2.6)	0.005
Hospital stay, median (range), day	5 (4–17)	10 (6–41)	<0.001
Start of walk, median (range), POD	0 (0–1)	1 (0–2)	<0.001
Start of drink, median (range), POD	1 (0–4)	1 (1–20)	NS
Start of diet, median (range), POD	2 (1–6)	3 (1–21)	<0.001

*POD* post-operative day, *Hb* hemoglobin, *RCC*-*LR* red cell concentrates-leukocytes reduced, *NS* not significant

^a^Non-steroidal anti-inflammatory drugs (NSAIDs), pentazocine hydrochloride, or fentanyl

### Hospital stay and resumption of normal activities

The median hospital stay in the ESDs was 5 days (range 4–17 days), compared with 10 days (6–41 days) in the LACs (*P* < 0.001). The median time for a patient who underwent ESD to start walking was POD 0 (range POD 0–1), compared with POD 1 (POD 0–2) in the LACs (*P* < 0.001). There was no significant difference in the median time for start of fluid intake between the two groups: POD 1 (range POD 0–4) in the ESD group and POD 1 (POD 1–20) in the LACs (*P* = NS). The median time for start of food intake was POD 2 (range POD 1–6) in the ESDs, and POD 3 (POD 1–21) in the LACs (*P* < 0.001) (Table 4).

### Intra- and postoperative complications

Intra- and postoperative complications were seen in 21 cases (7.0 %) in the ESDs, and in 28 cases (14.7 %) in the LACs (*P* = 0.005). Delayed bleeding and perforation associated with colorectal ESD was seen in 15 (5.0 %) and five (1.7 %) cases, respectively. All of the cases of delayed bleeding in the ESDs could be cured endoscopically. None of the patients in the ESDs needed a blood transfusion. With regard to perforation, four of five cases were cured by endoscopic clipping and ESD could also be completed. The other case had delayed perforation with acute generalized peritonitis and required emergency surgery. Diverting stoma due to postoperative adverse event was none in the ESDs. There were, in addition, no complications of intravenous anesthesia during ESD.

For major complications in the LACs, three patients (1.6 %) had delayed bleeding, four (2.1 %) had anastomotic leakage, and three (1.6 %) had peritonitis. For minor complication, three each (1.6 %) had wound infection or pneumonitis, two (1.1 %) had postoperative ileus, and one each (0.5 %) had wound dehiscence, subcutaneous hematoma, cholecystitis, paroxysmal atrial fibrillation, hives, or delirium. Three patients (1.6 %) required diverting stoma due to postoperative anastomotic leakage.

Temporal stoma was finally required in three of 16 cases in the ESDs in which additional LAC was performed because SM-d carcinoma or lymphovascular invasion in the lower rectum was confirmed by pathological investigation, while 20 cases (10.5 %) in the LACs required stoma (17 cases and three cases required temporal and permanent stoma, respectively) (*P* < 0.001) (Table 5).

**Table 5:** Clinical outcomes: safety (intra and postoperative complications and total cases of stoma)

	No. (%) ESDs (*n* = 300)	No. (%) LACs (*n* = 190)	*P* value
Total	21 (7.0)	28 (14.7)	0.005
Postoperative bleeding	15 (5.0)	3 (1.6)	
Perforation	5 (1.7)	–	
Anastomotic leakage	–	4 (2.1)	
Peritonitis	1 (0.3)	3 (1.6)	
Diverting stoma	0 (0)	3 (1.6)	
Ileus	0 (0)	2 (1.1)	
Surgical wound dehiscence	–	1 (0.5)	
Surgical wound infection	–	3 (1.6)	
Subcutaneous hematoma	–	1 (0.5)	
Pneumonitis	0 (0)	3 (1.6)	
Cholecystitis	0 (0)	1 (0.5)	
Abdominal incisional hernia	–	1 (0.5)	
Hives	0 (0)	1 (0.5)	
Paroxysmall atrial fibrillation	0 (0)	1 (0.5)	
Delirium	0 (0)	1 (0.5)	
Total cases of stoma	3 (1.0)	20 (10.5)	<0.001
Temporal/permanent, No	3/0	17/3	

These were consecutive patients with prospective data collection.

## Discussion

### Current status of colorectal ESD—compared with LAC

This is believed to be the first study to evaluate prospectively the clinical outcomes and perioperative clinical course in patients undergoing colorectal ESD and LAC. Simple comparison between ESD and LAC is problematic because each indication differs in Japan, as do procedures. ESD is used as a local treatment without lymph node dissection for adenoma, intramucosal or SM-s carcinomas, whereas LAC is performed for SM-d carcinomas and includes lymph node dissection. Worldwide, however, LAC is performed as the standard for lesions that are a good indication for colorectal ESD in Japan. The reasons are as follows: LAC is a low-invasive procedure compared with laparotomy and maintains QOL [23, 24]. Colorectal ESD, on the other hand, seems to be a difficult and hazardous procedure for non-expert endoscopists.

Endoscopic diagnosis of T stage in early cancer is also a hurdle and controversy, especially in western countries. High diagnostic accuracy of invasion depth of the lesion using magnifying colonoscopy, however, has been reported in Japan. ESD and LAC are selected for cT1a and cT1b, respectively, on the basis of preoperative diagnosis. Good clinical outcome and safety of colorectal ESD have been reported recently in Korea and Japan. Data on the perioperative clinical course and QOL evaluation of colorectal ESD, however, are scant at present. Postoperative QOL assessment for various diseases, in recent years, has been conducted and has become an important part of outcome assessment. The need for minimally invasive treatment to maintain patient QOL has increased. With further dissemination of colorectal ESD in the future, it will be crucial to establish its effectiveness and safety, as well as its minimal invasiveness compared with LAC. Although randomized controlled trials are ideal, their implementation is already difficult in Japan because the indications for colorectal ESD have been established and the technique has become popular.

### Characteristics of objective lesions and endoscopic diagnosis—in this study

Looking at the macroscopic type of lesions in this study, LST lesions accounted for 77 % in the ESD group. These lesions are large and difficult to treat by *en bloc* EMR, but it suggests that LSTs with the features of lateral growth type, which retain within SM-s invasion is an adaptive lesions for most of the colorectal ESD. In the LACs, on the other hand, depressed lesions accompanied with the feature of the likely SM-d invasion accounted for 42 %, even if the lesions were small in size.

Preoperative diagnosis of invasion depth based on conventional and magnifying colonoscopy is therefore important for the choice of ESD or LAC for early colorectal cancer [25]. Although endoscopic ultrasonography (EUS) is also a useful tool for evaluation of lesion depth, magnifying colonoscopy and EUS do not differ significantly for preoperative diagnosis of early colorectal cancer [26]. It is also a major advantage that magnifying colonoscopy can be used regardless of the size and location of the lesion immediately by just touching the zoom lever. In this study, there was a high rate of discrimination of lesion invasion depth by conventional and magnifying colonoscopy and treatment for early colorectal cancer could be selected adequately. If the preoperative diagnostic accuracy was poor and resulted in a high number of non-curative cases that underwent ESD, which then required additional LAC, ESD would not have been developed.

One of the two cases of ESD that had difficulty with histopathological evaluation was resected *en bloc* by ESD for local recurrence after TEM. In the other case, perforation occurred during ESD. Although endoscopic closure was achieved soon and the ESD procedure was completed, specimen collection was difficult. Adenoma was diagnosed by preoperative colonoscopy in this case and no recurrence was detected after 2.5 years.

Both of the LACs that had difficulty in histopathological evaluation had undergone EMR at another hospital. Although pathological evaluation showed that these cases had SM invasion after EMR, it was difficult to establish the precise depth of invasion according to the unknown orientation of the resected specimen and pVM. Six patients in the ESDs that were diagnosed pathologically as SM-d carcinoma did not receive additional LAC on request of the patients. One of these six patients did not visit our institution for the duration of follow-up. The remaining five cases did not experience any relapse after follow-up of 1.5–3.5 years.

### Effectiveness, less invasiveness, and safety—colorectal ESD

Median procedure time of colorectal ESD in this study was half that of LAC. In addition to shorter procedure time, colorectal ESD had high *en bloc* and curative resection rates. Colorectal ESD, therefore, demonstrated a high rate of effectiveness as a short-term clinical outcome.

With regard to perioperative clinical course, incidence of pyrexia (≥38 °C) and requirement for analgesic drugs were initially significantly lower for ESDs than LACs. This reflects the advantage of colorectal ESD; it can complete local resection of the intestinal, mucosal, and SM layers without laparotomy and interperitoneal procedures, including lymph node dissection.

We also investigated changes in WBC, CRP, and hemoglobin value between the preoperative period and POD 1. Variations in the inflammatory response indicators (WBC and CRP) were insignificant in the ESDs. Also, only 4 % of the ESD group had a ≥2 g/dl decrease in hemoglobin level. These absolute objective data indicate that ESD is less invasive than LAC. The reasons why the decrease in hemoglobin was low in the ESDs were the small amount of bleeding and immediate hemostasis during colorectal ESD.

Hospital stay in the ESDs was half that of the LACs. The time to resume fluid and food intake in the ESDs was POD 1 and POD 2, respectively. Although fluid intake in the ESDs started at almost the same time as in the LACs, food intake in the ESDs was significantly earlier. The median time to start walking was POD 0 in the ESDs. This indicated that patients could start walking soon after the procedure and this is one important factor associated with the lower level of invasiveness of colorectal ESD compared with LAC. Considering the lesser invasiveness of colorectal ESD, although hospital stay was 5 days in this study according to the clinical pathway which concerns the standardization of care process, day surgery may not be impossible [27].

The rate of major and minor complications with ESDs was 7.0 %. This was lower than with LACs, which is an established method of treatment worldwide. In four of five patients in the ESDs with perforation, complete closure was achieved utilizing an endoscopic clip (the other case required emergency ileo-cecal resection because of delayed perforation). A stable clinical course after the closure for perforation was achieved on the basis of conservative therapy such as fasting, hydration, and antibiotics. ESD has the following attributes for the management of perforation: (1) perforation occurs during the procedure, endoscopic closure using a clip can be achieved because the diameter of perforation is small compared to those by EMR; and (2) sufficient preparation can help the endoscopic management and prevent severe peritonitis.

The rate of delayed bleeding with ESD was higher than with LAC. Endoscopic hemostasis, however, could be achieved in all cases and no transfusions were required in the ESDs. In the LACs, there were three cases of delayed bleeding and two of them required blood transfusion; endoscopic and surgical hemostasis was required in one case each. The other case had a coexistent anastomotic leakage and required diverting stoma. Physical stress caused by delayed bleeding in ESD patients is lower than for LAC patients because it is only a minor factor and transfusion is not necessary in ESD. This is probably because ESD were conducted with coagulation of all exposed vessels in SM layer during and after the procedures.

Our results indicate the safety of colorectal ESD, because procedural complications such as delayed bleeding and perforation could be managed endoscopically, and there were no anesthesia complications. We should, however, pay attention to delayed perforation because emergency surgery is not preventable even though the incidence is quite low. Excess heating of endoscopic devices against the intestinal layer, which can lead to perforation, should be avoided so we prefer to use mainly bipolar devices and we should repair any damage to the muscularis layer by careful endoscopic clipping. These procedures are essential to prevent delayed perforation.

Stoma is associated with postoperative QOL but was only required in three cases (1.0 %), with additional LAC after ESD. In contrast, it was required in 20 cases (10.5 %) in the LACs. ESD enabled patients to maintain perioperative QOL with regard to anorectal function.

### Problem points and management guide of colorectal ESD

The technical difficulty and complications of ESD preclude the standardization of this novel procedure. Colorectal ESD, at present, is only practiced in Japan, Korea, and a few facilities in other countries. To overcome these obstacles, it is necessary to undergo ESD training using animal models and to accumulate experience of basic procedures such as endoscope insertion and EMR. The delay in applying ESD devices that are used exclusively in Japan is one of the hurdles in the dissemination of ESD overseas.

According to our study, rectal ESD is easier to perform than cecal ESD. The latter is likely to be difficult technically because the cecum is located in the innermost part of the large intestine and its wall is thin. The risk of perforation in the lower rectum is lower and there is better access for the endoscope and other devices. Rectal LAC is, paradoxically, more difficult because the pelvic cavity is anatomically narrow, surgical injuries tend to be more serious in lower rectal LAC. Temporary or permanent stomas are also necessary in some rectal LAC cases, despite the early stage nature of the lesions. Cecal LAC, in contrast, is easier to perform [21]. To decide on selection of treatment method (ESD or LAC), we should consider the following as well as preoperative endoscopic diagnosis: skill of the operator, reputation of the institution, and invasiveness for the patient. We should conduct diagnostic rectal ESD, to some extent aggressively, even if the lesion is all circumferential, or the differential diagnosis is difficult between SM-s and SM-d carcinoma by preoperative colonoscopy. We should, in contrast, select cecal LAC if cecal ESD is difficult to perform, in accordance with the endoscopist’s skill level.

Patient safety and accurate pathological assessment are important in terms of prognosis. If completion of ESD is difficult, we should stop the procedure and covert to LAC. This would enable us to avoid the complications of ESD and multiple piecemeal resection, which is associated with a high incidence of local recurrence or residual lesions [3]. Adequate additional surgery cannot be performed if the degree of SM invasion or lymphovascular invasion is suboptimal because of multiple piecemeal resection.

Our study had the following limitation. This was a single center study and the postoperative clinical course of colorectal ESD and LAC was based on the clinical pathway of our institution. Thus, a future multicenter study is required.

In conclusion, colorectal ESD is an effective technique with a short procedure time, high rate of curative resection, and procedural safety, as well as being less invasive than LAC. It is expected that colorectal ESD will continue to spread worldwide in the future with the development of endoscopic devices and simplification of treatment. Also, we should not forget that patients always request the less invasive, safe and adequate treatment.
